# Attention facilitates initiation of perceptual decision making: a combined psychophysical and electroencephalography study

**DOI:** 10.1007/s00221-024-06862-3

**Published:** 2024-05-30

**Authors:** Tomohiro Ueno, Hironori Kumano, Takanori Uka

**Affiliations:** Department of Integrative Physiology, Graduate School of Medicine, University of Yamanashi, 1110 Shimokato, Chuo-Shi, Yamanashi, Japan

**Keywords:** Human, Reaction time, Drift diffusion model, Parietal cortex

## Abstract

Humans can selectively process information and make decisions by directing their attention to desired locations in their daily lives. Numerous studies have shown that attention increases the rate of correct responses and shortens reaction time, and it has been hypothesized that this phenomenon is caused by an increase in sensitivity of the sensory signals to which attention is directed. The present study employed psychophysical methods and electroencephalography (EEG) to test the hypothesis that attention accelerates the onset of information accumulation. Participants were asked to discriminate the motion direction of one of two random dot kinematograms presented on the left and right sides of the visual field, one of which was cued by an arrow in 80% of the trials. The drift–diffusion model was applied to the percentage of correct responses and reaction times in the attended and unattended fields of view. Attention primarily increased sensory sensitivity and shortened the time unrelated to decision making. Next, we measured centroparietal positivity (CPP), an EEG measure associated with decision making, and found that CPP latency was shorter in attended trials than in unattended trials. These results suggest that attention not only increases sensory sensitivity but also accelerates the initiation of decision making.

## Introduction

By paying attention to objects, humans can selectively capture relevant information from the external environment and make decisions quickly and accurately. When the environment changes, the object of attention can be changed instantly allowing adaptive decisions to be made. Therefore, attention plays a critical role in adaptive behavior, but the neural mechanisms underlying this ability have not yet been completely elucidated.

Spatial attention is thought to be related to oculomotor areas, such as the frontal eye field (FEF) that is involved in saccades. Weak electrical stimulation of the FEF in monkeys such that the eyes do not move facilitates target detection (Moore and Fallah 2001). That is, stimulation of the oculomotor area leads to a state in which attention is directed to its response field. Moore and Armstrong (2003) also reported that electrical stimulation of the FEF during the recording of neurons in a sensory area, area V4, increased V4 activity when visual stimuli were presented in the V4 neuron receptive field corresponding to the FEF response field. Based on these studies, it was hypothesized that FEF activity increases the sensitivity of neurons in the sensory cortex, resulting in shorter reaction times.

On the other hand, the drift diffusion model (DDM) can be used to explain reaction time (Ratcliff 1978). The DDM is the most commonly used model to explain reaction time in two-alternative forced-choice tasks (Kelly et al. 2021). In the DDM, relevant information for a decision accumulates, and the decision is confirmed when it exceeds a certain threshold. If attention increases the sensitivity of sensory information, it is thought that information accumulates at a faster rate, but this has not been investigated via experiments.

Neural activity possibly related to the accumulation of information assumed in the DDM has been measured in the lateral intraparietal (LIP) area in monkeys (Roitman and Shadlen 2002; Gold and Shadlen 2007). When monkeys are trained to report the direction of motion, the speed (slope) of the LIP response increases with the intensity of the motion, peaking just before the saccade at all motion intensities (Roitman and Shadlen 2002). Recently, such decision-related activity has been measured in humans via electroencephalography (EEG) (O’Connell et al. 2012; Kelly and O’Connell 2013, 2015; Loughnane et al. 2016). This is captured as a positive wave in the parietal lobe, and has been designated centroparietal positivity (CPP) (O’Connell et al. 2012); CPP increases over time and peaks at the time of decision making (O’Connell et al. 2012; Kelly and O’Connell 2015). Thus, CPP might rise faster when attention is allocated.

In addition, attention may affect other components of the DDM to shorten reaction time. For example, attention may accelerate the initiation of decision making. Thus, the CPP might rise earlier when attention is allocated. Alternatively, it is possible that attention may lead to faster target selection and thus shorter reaction times. As the EEG component N2 is thought to be involved in target selection (Loughnane et al. 2016), N2 may be related to attention.

The present study examined how DDM parameters and human decision-related activity changes with and without attention based on the Posner task (Posner 1980). The Posner task is the most widely used task for studying spatial attention. In this task, subjects are asked to detect a target presented on a screen as quickly as possible, while a cue that tells the subject where the target is located is presented in advance. The target location matches the cue in approximately 80% of trials (cue valid condition), while the target location does not match the cue in approximately 20% of the trials (cue invalid condition). Reaction time tends to be prolonged in the latter condition. In this study, we combined the Posner task with the motion direction discrimination task, and used the DDM to determine whether sensory sensitivity and/or the time unrelated to decision making changes with the allocation of attention. We further measured EEG when attention was directed and not directed to clarify the effects of attention on CPP and N2.

## Materials and methods

This study was conducted in accordance with the Declaration of Helsinki and was approved by the Ethical Review Committee of the University of Yamanashi Graduate School of Medicine (No: 2137). The study population in the psychophysical experiment consisted of eighteen males (22–37 years old) and five females (22–27 years old). The study EEG experiment consisted of seventeen males (22–37 years old) and three females (22–27 years old), all of whom also participated in the psychophysical experiment. Written informed consent was obtained from all the subjects before the experiment.

### Psychophysical experiment

We combined the motion direction discrimination task and the Posner task to examine factors that reduce reaction time when attention is directed.

In the psychophysical experiment, participants sat in an electromagnetic shield room covered with a blackout curtain facing a monitor (VIEWPixx 3D Lite; VPixx Technologies) placed 57 cm from the eyes and placed their chin on a chin rest to maintain a constant distance from the monitor. A keyboard was placed in front of the participants at a height that was comfortable to allow them to press a key. The monitor was 53.1° wide and 29.9° high and had resolution of 1920 × 1080 pixels with a vertical synchronization frequency (refresh rate) of 120 Hz. The task was run using MATLAB (R2007a) with the psychtoolbox-3 (Brainard 1997) on a Macintosh computer running OS10.

**Fig. 1:**
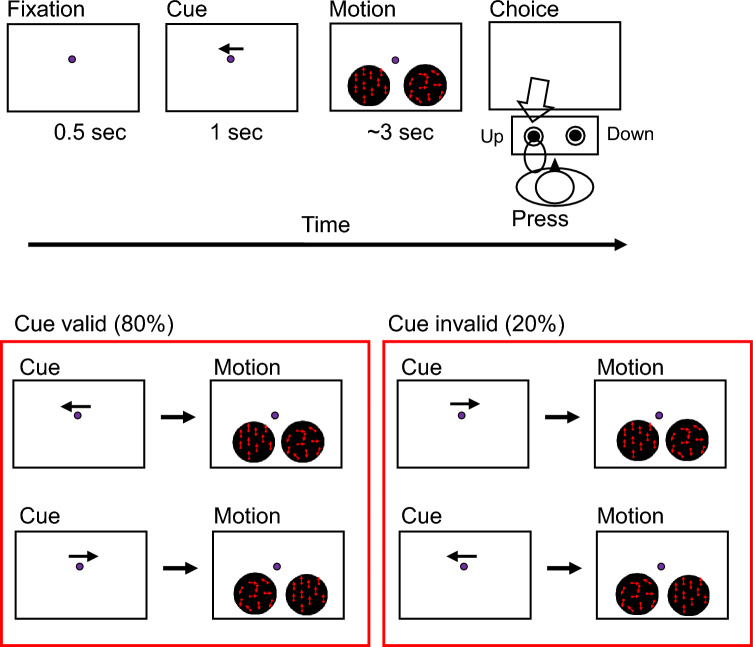
Attention task (psychophysical experiment). Participants were instructed to focus their attention on one of two targets and to press a button quickly and accurately to report the direction of motion of the target. The direction of the arrow and the position of the moving target were matched in 80% of cases, and they were not matched in 20% of cases

Participants fixated on a fixation point in the center of the monitor (Fig. 1). An arrow appeared in the upper center of the monitor for 1 s, and then a random dot appeared within a circular aperture on each side. Motion strength was modulated by manipulating the percentage of coherently moving dots (motion coherence). Some dots on either side (target) moved either up or down, while the dots on the other side had random motion (0% coherence). The direction of the arrows was adjusted so that approximately 80% of the arrows and target coincided (cue valid condition) and 20% of the arrows and target did not coincide (cue invalid condition). The participants were told verbally that the arrow and target positions did not match in some cases. Each participant performed five trials for 28 conditions: 2 target positions (right or left), 2 directions of dot motion (up or down), and 7 coherences (1%, 3%, 6%, 12%, 24%, 48%, 96%) per block for a total of 140 trials/block; they also performed five blocks with a rest between blocks for a total of 700 trials. As the coincidence of the arrow and target was randomly selected for each trial, approximately 560 trials were cue valid and 140 trials were cue invalid for each participant. The conditions and trial numbers are summarized in Table 1. The task was initiated by pressing the keyboard, with the correct responses consisting of pressing “F” if the dot moved upward or “J” if the dot moved downward. When the subject pressed the button, the dot disappeared immediately, providing feedback regarding whether the answer was correct or incorrect.

**Table 1:** Conditions and trial numbers for each experiment. Trial numbers for cue valid and cue invalid conditions are approximate numbers because the coincidence of the arrow and target was randomly selected for each trial (i.e. cue valid and cue invalid conditions were randomly selected).

Psychophysics	Coherences(%)	Trials/coherence	Trials/block	Total trials
Cue valid	1.5, 3, 6, 12, 24, 48, 96	16	～112	～560
Cue invalid	1.5, 3, 6, 12, 24, 48, 96	～4	～28	～140
Total	7 coherences	20	140	700

EEG	Coherences(%)	Trials/coherence	Trials/block	Total trials
Cue valid	35, 70	～32	～64	～320
Cue invalid	35, 70	～8	～16	～80
Total	2 coherences	40	80	400

### EEG experiment

**Fig. 2:**
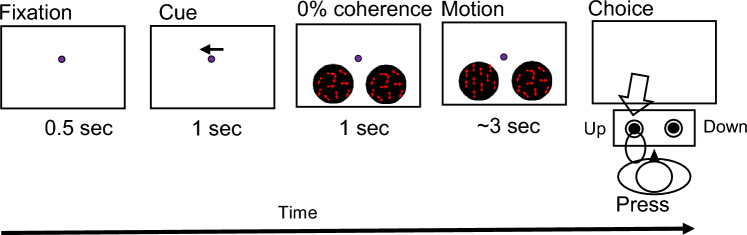
Attention task (EEG experiment). Participants performed the same task as in the psychophysical experiment. However, the arrow at the center of the monitor appeared for 1 s, and the left and right random dots moved randomly (0% coherence) at first. Then, either the left or right dots moved up or down (coherence: 35% or 70%) for 3 s

In the EEG experiment, participants were asked to perform a similar task under the same environmental conditions as in the psychophysical experiment. However, after the arrow (right or left) in the center of the monitor appeared for 1 s, the left and right random dots initially moved randomly (0% coherence), and after another 1 s, a portion of either the left or right dots (coherence: 35% or 70%) moved either up or down for 3 s (Kelly and O’Connell 2013; Loughnane et al. 2016; Newman et al. 2016) (Fig. 2). This was done so that the transient visual evoked potentials (VEPs) at random dot onset did not overlap with the EEG signals at the onset of dot motion (Kelly and O’Connell 2013). Each participant performed 10 trials under 8 conditions: 2 coherences (35% or 70%), 2 target positions (right or left), and 2 directions of dot motion (up or down) per block for a total of 80 trials/block; they also performed five blocks with a rest between blocks for a total of 400 trials. As the coincidence of the arrow and target was randomly selected for each trial, roughly 320 trials were cue valid and 80 trials were cue invalid for each participant. The conditions and trial numbers are summarized in Table 1. As the disappearance of the random dots would evoke transient EEG responses, the dots did not disappear when the participant pressed the button but continued to be presented for 3 s.

EEG measurements were made using a digital DC electroencephalograph (BIO-NVX36; Medical Computer Systems), and recordings were made using a NeoRec software. EEG measurements were taken at 19 points (International 10–20 method) using an EEG cap. The resistance between the head surface and the electrodes was adjusted to keep it below 10 kΩ. Reference electrodes were A1/A2, and recordings were made at 2000 Hz. The EEG measurements were bandpass filtered at 0.05–70 Hz (notch filter: 50 Hz) and the EEG input from each electrode was digitized and stored in EDF + file format.

### Analysis

#### Psychophysical analysis

**Fig. 3:**
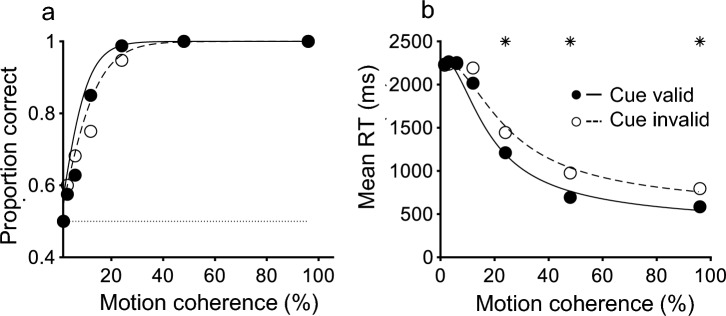
Results of the psychophysical experiment from one participant (700 trials). **a** The horizontal axis represents motion coherence and the vertical axis represents the percentage of correct responses. The solid line represents the cue valid condition and the dotted line represents the cue invalid condition, which was fit to the DDM. **b** The horizontal axis represents motion coherence and the vertical axis represents reaction time. The solid line represents the cue valid condition and the dotted line represents the cue invalid condition, which was fit to the DDM. Statistically significant differences between the cue valid and cue invalid conditions (Wilcoxon rank sum test, *P* < 0.05) are plotted with an asterisk at the top of the figure

From the behavioral data obtained, we calculated the reaction time and accuracy separately for the cue valid and cue invalid conditions at each coherence. Next, we approximated the relations between reaction time and accuracy separately for each condition using the DDM (Fig. 3).

The following equation was used for fitting in the DDM (Palmer et al. 2005).

1 $$\text{Pc}\left(\text{coh}\right)=\frac{1}{1+\text{exp}\left(-2\text{A}\bullet \text{K}\bullet \text{coh}\right)}$$

2 $$\text{T}\left(\text{coh}\right)=\frac{\text{A}}{\text{K}\bullet \text{coh}}\text{tanh}\left(\text{A}\bullet \text{K}\bullet \text{coh}\right)+{\text{t}}_{\text{R}}$$

where Pc is proportion correct, coh is motion coherence, A is threshold, K is sensitivity, T is reaction time, and t_R_ is non-decision time. t_R_ was constrained to be larger than 200 ms.

To examine whether the threshold (A), sensitivity (K), and non-decision time (t_R_) were significantly different between the conditions, we used a six-parameter DDM and the Akaike Information Criterion (AIC) (Akaike 1974). The six parameters were A, δA, K, δK, t_R_, and δt_R_. The parameters were A, K, and t_R_ when only the cue invalid condition was fit, and the parameters for the cue valid condition were threshold (A + δA), sensitivity (K + δK), and non-decision time (t_R_ + δt_R_). The AIC of the full six-parameter model was compared to the AIC of five-parameter DDMs that did not use either δA, δK, or δt_R_. If the AIC of the five-parameter model was higher than the AIC of the six-parameter model, then the removed δ parameter was determined to be different from 0.

#### EEG analysis

EEG data were bandpass filtered at 1–20 Hz. For each condition (35% coherence: cue valid or cue invalid; 70% coherence: cue valid or cue invalid), the average waveform was calculated for an epoch starting 1 s before and ending 4 s after the dot began to move. CPP was calculated by subtracting the average waveform of the surrounding area from the average waveform of the Cz and Pz electrodes.

The slope of the CPP was obtained by linear regression of the CPP from 200 to 400 ms after the random dots began moving for each participant. For CPP latency, the number of trials in the cue valid condition was downsampled to match the number of trials in the cue invalid condition by randomly selecting among trials without overlap. This is because the variability of EEG signals depends on the number of trials. The baseline standard deviation (SD) of CPP from –250 to 0 ms was calculated, and latency was determined as the time when CPP first exceeded 3 SD after 150 ms from the start of random dot motion. In the cue valid condition, the random selection of trials was performed 10,000 times, such that 10,000 CPP latencies were calculated. The median of the distribution of latencies was used as the final latency.

N2 was calculated from the T5/T6 electrodes. N2 amplitude was determined as the minimum value between 0 and 500 ms after the random dots began moving. The N2 latency was determined as the time when N2 reached the minimum value. MATLAB Ver2020a was used for all analyses.

## Results

### Psychophysical results

We conducted 16,100 trials with 23 participants. Figure 3 compares the performance of the motion direction discrimination task in the cue valid and cue invalid conditions for one participant. Panel (a) shows the psychometric functions, with the smooth curve representing the DDM fit (Eqs. 1 and 2) (Palmer et al. 2005). As expected, the percentage of correct responses was higher in the cue valid condition than in the cue invalid condition. Panel (b) shows the chronometric functions, and as expected, reaction time was faster in the cue valid condition than in the cue invalid condition. These results are consistent with previous studies (Posner 1980; Meiran 1996).

Next, the parameters of the DDM were carefully examined to analyze the factors that contribute to shortening of the reaction time under the cue valid condition. Three parameters were used in the DDM: A (threshold), K (sensitivity), and t_R_ (non-decision time). In principle, the shortening of reaction time can be explained by a decrease in A, an increase in K, or a shortening of t_R_.

**Fig. 4:**
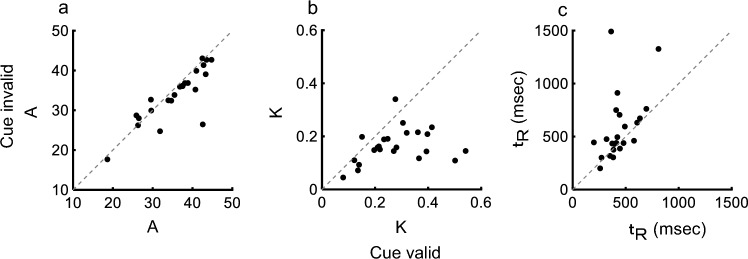
Comparison of DDM parameters across 23 participants. **a** A (threshold), **b** K (sensitivity). **c** t_R_ (non-decision time)

Figure 4 shows a comparison of the three parameters for the cue valid and cue invalid conditions across 23 individuals. The median threshold (A) was larger (cue valid: 37.5, cue invalid: 35.2; Wilcoxson signed-rank test, *P* = 0.016), the median sensitivity (K) was larger (cue valid: 0.270, cue invalid: 0.158; Wilcoxson signed-rank test, p < 0.001) and the median non-decision time (t_R_) was shorter (cue valid: 412, cue invalid: 461; Wilcoxson signed-rank test, *P* = 0.024) for the cue valid condition. Therefore, across participants, the increase in K and shortening of t_R_ contributed to shortening of the reaction time under the cue valid condition: the larger A in the cue valid condition cannot explain shortening of reaction time.

We also used the AIC to evaluate which parameter contributed to the shortening of reaction time. Here, A, K, and t_R_ were used in the cue invalid condition, and δA, δK, and δt_R_ were added to these three parameters in the cue valid condition for a total of six parameters for the DDM fit. Because it was difficult to fit the six-parameter model for each individual data, we combined the data across participants, before performing the fit.

To examine which δ parameters were different from 0, we compared the AIC of the six-parameter model with all δ parameters included and five-parameter models without each δ parameter. The AIC for the full model (six-parameter model) was 646, whereas the AIC was 661 when δt_R_ was excluded, 653 when δK was excluded, and 711 when δA was excluded. Therefore, as the AIC was greater than the AIC of the full model when any δ parameter was excluded, we concluded that the error was greater without each δ parameter. However, as noted above, the larger A in the cue valid condition cannot explain shortening of reaction time, suggesting that the increase in sensitivity and the shortening of non-decision time were the main contributors to the shortening of reaction time.

### Results of EEG measurements

Based on the results of the psychophysical experiments, we expected that sensitivity would increase and non-decision time would decrease with direction of attention. Here, we examined these two events from EEG measurements of decision-related activity. The EEG measure used was CPP, which is a decision-related activity with a coherence-dependent rise. As CPP may be overridden by the VEP, either the left or right dots were moved after presentation of dots that moved in a random direction (0% coherence) (Loughnane et al. 2016). Data from a total of 8000 trials were obtained from twenty participants.

### CPP slope increased and latency decreased with attention

**Fig. 5:**
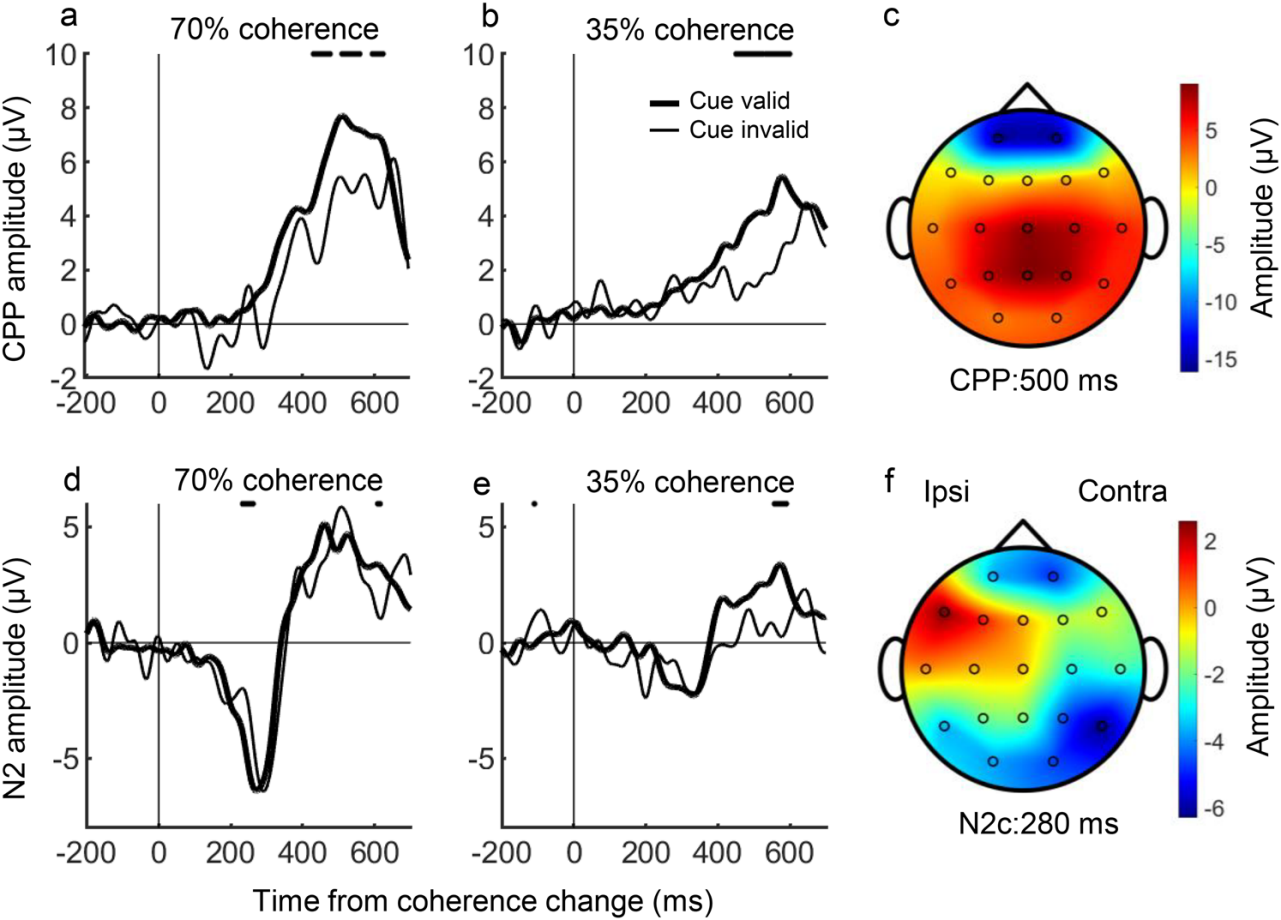
Centroparietal positivity (CPP) and N2c from one participant (400 trials). **a** CPP for 70% coherence trials. **b** CPP for 35% coherence trials. **c** Topography of CPP using the 70% coherence cue valid condition. **d** N2c for 70% coherence trials. **e** N2c for 35% coherence trials. **f** Topography of N2c using the 70% coherence cue valid condition. The horizontal axis in a, b, d, e represents the time from motion coherence change and the vertical axis represents the EEG potential. The thick line represents the cue valid condition and the thin line represents the cue invalid condition. Statistically significant differences between the cue valid and cue invalid conditions (Wilcoxon rank sum test, *p* < 0.05) are plotted as a line at the top of the figure

**Fig. 6:**
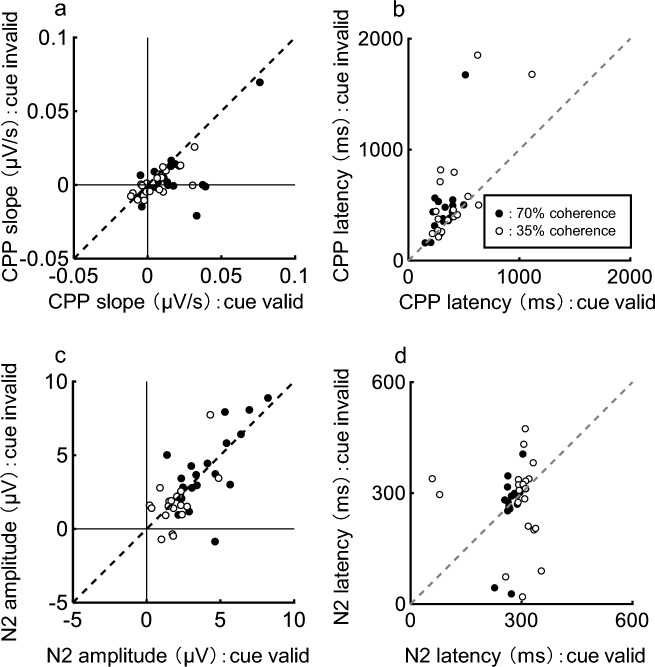
Comparison of **a** CPP slope, **b** CPP latency, **c** N2c amplitude and **d** N2c latency. Black circles, 70% coherence; white circles, 35% coherence

First, we analyzed how the CPP slope changed with attention. Figure 5a and b shows the CPP aligned at the beginning of dot motion for a typical participant. CPP topography is shown in Fig. 5c, which is consistent with a previous study (Loughnane et al. 2016). The CPP increased more quickly with a higher coherence and more slowly with a lower coherence. CPP slope was steeper in the cue valid condition compared to the cue invalid condition. To examine whether the CPP slope differed significantly between the cue valid and invalid conditions, the CPP slope for all participants was measured in the interval of 200–400 ms after the dot began to move and compared using Wilcoxon’s signed-rank test across conditions. There was a significant difference between the cue valid and cue invalid conditions at 70% coherence (*P* = 0.0019) and 35% coherence (*P* = 0.021) (Fig. 6a). Therefore, the CPP slope increased with attention.

Next, we analyzed how the CPP latency changed with attention. Figure 5a and b shows that CPP started to rise more quickly in the cue valid condition compared to the cue invalid condition. Figure 6b compares the CPP latency for twenty participants in the cue valid and cue invalid conditions. Wilcoxon’s signed-rank test confirmed that CPP latency was shorter for the cue valid compared to the cue invalid condition in both 70% (*P* = 0.0012) and 35% (*P* = 0.031) coherences. Therefore, the CPP latency was shortened by attention.

### Amplitude and latency of N2 were not altered by attention

Previous studies have shown that N2 is a negative wave with a latency of approximately 200 ms in the lateral occipital/temporal cortex, which responds to visual stimuli and is associated with target selection (Loughnane et al. 2016). Here, we examined whether the amplitude and latency of N2 differed between the cue valid and cue invalid conditions. We focused on the N2 responses to the contralateral visual field stimuli (N2c) because N2 responses to the ipsilateral visual field stimuli (N2i) were very small in amplitude.

Figure 5d and e shows N2c in the cue valid and cue invalid conditions for a typical participant. N2c topography is shown in Fig. 5f, which is also consistent with a previous study (Loughnane et al. 2016). There were few differences in amplitude and latency between conditions in either coherence. To examine whether the amplitude and latency of N2c differed significantly between conditions, we compared the N2c between the cue valid and cue invalid conditions using Wilcoxon’s signed-rank test (Fig. 6c and d). The N2c amplitude was not significantly different for the 70% (*P* = 0.55) or 35% (*P* = 0.77) coherence stimuli between the cue valid and cue invalid conditions. The N2c latency was also not significantly different between the 70% (*P* = 0.22) and 35% (*P* = 0.91) coherence stimuli between the cue valid and cue invalid conditions. Therefore, there were no differences in N2c amplitude and latency between the cue valid and cue invalid conditions.

## Discussion

In the present study, our psychophysical study showed that attention enhanced sensory sensitivity and reduced the amount of time spent outside of decision making. In addition, our EEG study showed that CPP, a decision-related neural signal increased its slope and shortened its latency with attention. Attention has been suggested to increase the sensory sensitivity of the spatial region to which attention is directed (Moore and Zirnsak 2017). Indeed, weak electrical stimulation of the FEF increases the gain of sensory information in V4 neurons (Moore and Armstrong 2003). Taken together, the results of previous studies and those presented here suggest that attention, in addition to changes in sensitivity, also speeds up decision initiation. Top-down signals from the FEF may control not only sensory area sensitivity but also decision onset in a visual field-specific manner.

### Evaluation of the DDM parameters

In this study, in addition to an increase in sensitivity, we found a shortening of non-decision time and an increase of the decision threshold with attention. The increase of decision threshold may be due to our task design. When motion coherence was low, it was difficult to determine which aperture (right or left) contained coherent motion. Thus, participants had to guess, resulting in similar reaction times for cue valid and cue invalid conditions. This would lead to an underestimation of threshold (A) in the cue invalid condition, because A is related to the difference in reaction time between easy and difficult conditions (Palmer et al. 2005), which is smaller in the cue invalid condition.

The shortening of non-decision time (t_R_), conversely, is mainly related to reaction time in the easy conditions. Even at 96% coherence, we found, on average, a 220 ms difference between cue valid and invalid conditions (Fig. 3b), which is larger than the difference in non-decision time (t_R_) between the cue valid and cue invalid conditions. This may be because t_R_ was not well constrained, especially since the reaction time did not reach the floor under the cue invalid condition. Nonetheless, t_R_ was shorter in the cue valid condition.

### Measurements of CPP and N2c

In our task, the cue valid condition necessarily contained more trials than the cue invalid condition. Thus, estimates of CPP and N2c parameters were less reliable in the cue invalid condition. However, we believe that this does not explain the increase in CPP slope in the cue valid condition. It is possible that added trials may reveal a difference in N2c amplitude and latency between conditions.

Calculation of CPP latency is dependent on trial number. This is because averaging more trials will lead to less noise and a smaller standard deviation for the baseline, which we used as a reference for calculating latency. A smaller baseline standard deviation will lead to increased detection sensitivity, and thus a shortening of CPP latency. To overcome this issue, we downsampled the number of trials for the cue valid condition before calculating CPP latency, so that the cue valid and invalid conditions had the same number of trials for each participant. Despite this adjustment, CPP latency was shorter in the cue valid condition, suggesting that decision onset was accelerated in the cue valid condition.

### Relation between CPP and N2

The CPP is an EEG signal that provides insight into human decision making (O’Connell et al. 2012). This signal is similar to that found in monkeys (Gold and Shadlen 2007) and may be linked to the timing of decision making regardless of the characteristics of the visual stimulus (O’Connell et al. 2012). On the other hand, N2 is active during target selection, such as in visual search tasks (Loughnane et al. 2016).

Loughnane et al. (2016) examined the relations between N2 and CPP using stimuli in which either of the two targets moved, and reported that the N2c latency was correlated with the latency and slope of CPP. From this, they concluded that N2c influences CPP in ambiguous situations where it is not known which of the two targets moves. In the present study, the direct relation between N2c and CPP was difficult to scrutinize due to the small number of trials. However, the amplitude and latency of N2c were unaffected by attention, whereas the CPP slope was larger and the CPP latency was shorter with than without attention. Therefore, it is unlikely that CPP was affected by N2c in this study. N2c may not affect CPP in situations where target location is unambiguous, as in this study, i.e., the participants knew in advance which targets would move 80% of the time. This is consistent with the top-down hypothesis of attention (Moore and Zirnsak 2017). In top-down attention, oculomotor areas, such as the FEF, control target selection. Therefore, it is not surprising that phenomena such as the faster increase in CPP when attention was directed, as observed in the present study, can be explained by top-down attention and that CPP is unaffected by N2c. On the other hand, in Loughnane et al. (2016), there was no top-down control and the targets had to be selected in a bottom-up manner, which may have led to N2c controlling the latency of CPP. Taken together, these observations suggest that N2 is locked to the physical timing of the stimulus, independent of attention, and changes in amplitude may depend on stimulus strength (saliency) (Loughnane et al. 2016). Regardless of whether or not attention is directed, visual information is expected to reach the temporal lobe in the same way if the stimulus is relevant to the performance of the task. How that information is used to make decisions may then be controlled by another higher brain region.

### Other psychophysical effects that may be explained by a change in onset of decision making

Many studies have shown that a warning signal given before the stimulus is applied shortens the reaction time (e.g., Pekka and Risto 1981). This type of warning signal may hasten the initiation of the decision-making process, as in our study.

Otherwise, in experiments using a switching task, researchers have compared reaction times when repeating a task to those when switching from one task to another: reaction times increased in trials in which the task was switched (e.g., Meiran 1996). One cause of this switch cost may be a delay in initiating the decision-making process. In fact, in one study, systemic administration of ketamine to monkeys performing a switching task increased reaction time and delayed the onset of decision making (Suda and Uka 2022). These observations suggest that the decrease in reaction time may be due to delayed decision initiation and that NMDA receptors may be involved in the control of decision initiation.

### Attentional dysfunction and its relevance to this study

Umarova et al. (2011) performed a target detection task using the Posner task in patients with hemispatial neglect. They reported that patients with hemispatial neglect had lower detection rates and longer reaction times on both the healthy and affected sides compared to healthy subjects, with particularly lower detection rates and longer reaction times on the affected side. Corbetta et al. (2005) also combined the Posner task with fMRI to study the relations between detection rate and reaction time during the acute and chronic phases of hemispatial neglect. In patients with hemispatial neglect, top-down signals from the frontoparietal to occipital lobes were weaker and both responses to visual stimuli and spatial selectivity of the left and right hemispheres were reduced in the acute phase compared to the chronic phase (Corbetta et al. 2005). As a result, the detection rate of stimuli presented in the left visual field decreased, while in the chronic phase the detection rate and reaction time became faster as top-down signals from the dorsolateral parietal cortex improved (Corbetta et al. 2005).

We speculated that if the task used in the present study had been applied in patients with hemispatial neglect, as reported by Corbetta et al. (2005), the top-down signal from the frontoparietal lobe would be weaker, resulting in slower decision initiation and prolonged CPP latency. With regard to N2, it is also assumed that higher attentional centers modulate bottom-up signals, as reported by Umarova (2011). Therefore, it is expected that the N2 latency may be longer and its amplitude may be attenuated in patients with hemispatial spatial neglect.

In summary, spatial attention not only affects the sensitivity of information accumulation, but also control the onset of information accumulation. Further studies can address the neural mechanism of decision onset by analyzing LIP responses in monkeys.

## Data Availability

The data that support the findings of this study are available from the corresponding author upon reasonable request.

## References

[CR1] Akaike H (1974). A new look at the statistical model identification. IEEE Trans Automat Control.

[CR2] Brainard DH (1997). The psychophysics toolbox. Spat Vis.

[CR3] Corbetta M, Kincade MJ, Lewis C, Snyder AZ, Sapir A (2005). Neural basis and recovery of spatial attention deficits in spatial neglect. Nat Neurosci.

[CR4] Gold JI, Shadlen MN (2007). The neural basis of decision making. Annu Rev Neurosci.

[CR5] Kelly SP, O’Connell RG (2013). Internal and external influences on the rate of sensory evidence accumulation in the human brain. J Neurosci.

[CR6] Kelly SP, O’Connell RG (2015). The neural processes underlying perceptual decision making in humans: recent progress and future directions. J Physiol.

[CR7] Kelly SP, Corbett EA, O’Connell RG (2021). Neurocomputational mechanisms of prior-informed perceptual decision-making in humans. Nat Hum Behav.

[CR8] Loughnane GM, Newman DM, Bellgrove MA, Lalor EC, Kelly SP, O’Connell RG (2016). Target selection signals influence perceptual decisions by modulating the onset and rate of evidence accumulation. Curr Biol.

[CR9] Meiran N (1996). Reconfiguration of processing mode to task performance. J Exp Psychol.

[CR10] Moore T, Armstrong KM (2003). Selective gating of visual signals by microstimulation of frontal cortex. Nature.

[CR11] Moore T, Fallah M (2001). Control of eye movements and spatial attention. Proc Natl Acad Sci USA.

[CR12] Moore T, Zirnsak M (2017). Neural mechanisms of selective visual attention. Annu Rev Psychol.

[CR13] Newman DP, Lockley SW, Loughnane GM, Martins AP, Abe R, Zoratti MTR, Kelly SP, O’Neill MH, Rajaratnam SMW, O’Connell RG, Bellgrove MA (2016). Ocular exposure to blue-enriched light has an asymmetric influence on neural activity and spatial attention. Sci Rep.

[CR14] O’Connell RG, Dockree PM, Kelly SP (2012). A supramodal accumulation-to-bound signal that determines perceptual decisions in humans. Nat Neurosci.

[CR15] Palmer J, Huk AC, Shadlen MN (2005). The effect of stimulus strength on the speed and accuracy of a perceptual decision. J Vision.

[CR16] Pekka N, Risto N (1981). Foreperiod and simple reaction time. Psychological.

[CR17] Posner MI (1980). Orienting of attention. J Exp Psychol.

[CR18] Ratcliff R (1978). A theory of memory retrieval. Psychol Rev.

[CR19] Roitman JD, Shadlen MN (2002). Response of neurons in the lateral intraparietal area during a combined visual discrimination reaction time task. J Neurosci.

[CR20] Suda Y, Uka T (2022). The NMDA receptor antagonist ketamine impairs and delays context-dependent decision making in the parietal cortex. Commun Biol.

[CR21] Umarova RM, Saur D, Kaller CP, Vry MS, Glauche V, Mader I, Hennig J, Weiller C (2011). Acute visual neglect and extinction: distinct functional state of the visuospatial attention system. Brain.

